# Formulation of Orally Disintegrating Films as an Amorphous Solid Solution of a Poorly Water-Soluble Drug

**DOI:** 10.3390/membranes10120376

**Published:** 2020-11-27

**Authors:** Pattaraporn Panraksa, Pratchaya Tipduangta, Kittisak Jantanasakulwong, Pensak Jantrawut

**Affiliations:** 1 Department of Pharmaceutical Sciences, Faculty of Pharmacy, Chiang Mai University, Chiang Mai 50200, Thailand; pattaraporn.prs@gmail.com (P.P.); ptipduangta@gmail.com (P.T.); 2 Division of Packaging Technology, School of Agro-Industry, Faculty of Agro-Industry, Chiang Mai University, Chiang Mai 50100, Thailand; jantanasakulwong.k@gmail.com; 3 Cluster of Agro Bio-Circular-Green Industry (Agro BCG), Chiang Mai University, Chiang Mai 50100, Thailand

**Keywords:** phenytoin, poorly water-soluble drug, orally disintegrating films, cosolvent, amorphous solid dispersion

## Abstract

The objective of the present study was to develop an orally disintegrating film (ODF) for a poorly water-soluble drug, phenytoin (PHT), using the cosolvent solubilization technique to achieve the amorphization of the drug, followed by the preparation of ODFs. Eleven formulations were prepared with different polymers, such as polyvinyl alcohol (PVA) and high methoxyl pectin (HMP) by the solvent casting method. The prepared films were subjected to characterization for weight variations, thickness, surface pH, disintegration time and mechanical strength properties. Then, differential scanning calorimetry, X-ray diffraction analysis and the drug release patterns of the selected films were evaluated. Among the prepared formulations, the formulation composed of 1% *w*/*w* of PVA, 0.04% *w*/*w* of sodium starch glycolate with polyethylene glycol 400, glycerin and water as cosolvents (PVA-S4) showed promising results. The physical appearance and mechanical strength properties were found to be good. The PVA-S4 film was clear and colorless with a smooth surface. The surface pH was found to be around 7.47 and the in vitro disintegration time was around 1.44 min. The drug content of the PVA-S4 film was 100.27%. X-ray diffractometry and thermal analysis confirmed the transition of phenytoin in the PVA-S4 film into a partially amorphous state during film preparation using the cosolvent solubilization approach. The resulting PVA-S4 film showed a higher dissolution rate in comparison to the film without a cosolvent. Overall, this study indicated the influence of cosolvents on enhancing the solubility of a poorly water-soluble drug and its film dissolution.

## 1. Introduction

Epilepsy is one of the most serious neurological diseases and affects more than 70 million people worldwide [1]. It is a brain disorder in which the neuronal networks’ electrical activity in the cerebral cortex becomes hypersynchronous, leading to seizures [2]. Delayed treatment for epileptic seizures can lead to a longer seizure duration, an increased frequency of hypotension and higher mortality rate [3]. Thus, the treatment of epilepsy mainly focuses on stopping a seizure with the correct medication once it has started without producing any unacceptable side effects [4]. Antiepileptic drugs are the main type of medication used for the treatment of acute and chronic epilepsy. Phenytoin is indicated to control generalized tonic–clonic (grand mal) and complex partial (psychomotor, temporal lobe) seizures, and to prevent and treat seizures that occur during or following neurosurgery [5]. There are many available dosage forms of phenytoin in the market, such as oral suspensions, chewable tablets, capsules and intravenous injections. However, some researchers have directed their research activity to the reformulation of existing drugs into new dosage forms. One such relatively new dosage form is the orally disintegrating film (ODF)—a thin film, which is prepared using hydrophilic polymers that rapidly dissolve on the tongue or buccal cavity [6,7]. This ODF delivery system has numerous advantages over conventional dosage forms (e.g., tablet, capsule and intravenous injection), such as convenience, accurate dosing, rapid onset of action with increased bioavailability, due to bypassing the hepatic first-pass effect and drug delivery without pain [6]. Moreover, ODFs can be used for dysphasic and schizophrenic patients and are taken without water, due to their ability to disintegrate within a few minutes, releasing medication in the mouth. There are still no official guidelines available to determine a disintegration time or specify a time limit for the disintegration of ODFs. However, according to the European Pharmacopoeia (Ph. Eur.) and British Pharmacopoeia (BP) regarding the disintegration time of orally disintegrating tablets (ODTs, which may be applied to ODFs), a time limit of 3 min for disintegration is specified [8]. Various approaches are employed to formulate ODFs, such as solvent casting, semisolid casting, hot-melt extrusion, solid dispersion extrusion and rolling [9]; of these, solvent casting is frequently used. A variety of polymers such as hydroxypropyl methylcellulose (HPMC) E5, polyethylene oxide (PEO), polyvinyl alcohol (PVA) and polyvinyl pyrrolidone (PVP) are available for the preparation of oral films. A polymer can be used alone or in combination to obtain the desired ODF properties. For example, in a study by Mashru et al. [10], orally disintegrating PVA films of salbutamol sulfate were successfully developed using the solvent casting method. The PVA films exhibited good mechanical characteristics and showed a rapid release of 90% in the first 2 min. In another study by Newton et al. [11], fast disintegrating oral hybrid films of propranolol hydrochloride were successfully prepared using pectin and other synthetic polymers such as HPMC, HPMC E15 LV, PVA and Carbopol. All films showed complete drug release at pH 6.8 within 12 min, and their mouth dissolving time and dissolution time remained relatively stable after three months. To overcome the solubility problem of poorly water-soluble drugs, many approaches such as cosolvency, solid dispersion, self-microemulsifying drug delivery systems (SMEDDS), salt formation and others have been developed [12]. However, there are limited studies on the preparation of amorphous ODFs loaded with phenytoin using the solubility enhancement technique to achieve the amorphization of the drug.

Sodium starch glycolate (SSG), which was selected as a superdisintegrant in this study, is the sodium salt of cross-linked carboxymethylated starch. It is classified as a superdisintegrant due to its very strong swelling characteristics [13]. SSG is added to solid dosage form formulations to promote the breakup of the dosage form into smaller fragments in an aqueous environment, thereby increasing the available surface area and promoting a more rapid release of the drug substance. In a previous study by Yellanki et al. [14], phenobarbital mouth-dissolving films were developed, and the SSG was used as a superdisintegrant. The results showed that, of all the developed films, the films consisting of SSG in a concentration of 1% *w*/*v* showed a good disintegration and dissolution time and showed an improved dissolution when compared with the available marketed films.

Thus, this present investigation was aimed at the formulation of orally disintegrating films containing a poorly water-soluble drug, using a phenytoin base as a model drug, by the solvent casting method using PVA and high methoxyl pectin (HMP) as polymers, as well as the evaluation of their physicochemical properties and in vitro dissolution profile, which may produce a rapid onset of action.

## 2. Materials and Methods

### 2.1. Materials

Phenytoin (PHT) (m.w. 252.27 g/mol, logP octanol/water 2.47, water solubility 32 mg/L at 22 °C [15]) was purchased from Merck (Darmstadt, Germany). Polyvinyl alcohol (PVA, 23–27 mPa∙s, 4% in H_2_O (20 °C); 87.0%–89.0% degree of hydrolysis) was purchased from Japan Vam & Poval Co., Ltd. (Osaka, Japan). High methoxyl pectin (HMP, degree of esterification = 60%) was purchased from Du Pont^®®^ (Wilmington, DE, USA). Polyethylene glycol (PEG) 400 was purchased from NOF Corporation (Tokyo, Japan). Refined glycerin was purchased from Srichand United Dispensary Co., Ltd. (Bangkok, Thailand). Ethanol was purchased from Bangkok Alcohol Industrial Co., Ltd. (Bangkok, Thailand). Sodium starch glycolate (SSG) was purchased from JRS Pharma (Rosenberg, Germany). Distilled water served as the solvent for preparing film solutions. All of the other reagents were of analytical grade.

### 2.2. Cosolvency

Solubility studies of phenytoin in mixed-solvent systems were carried out by the equilibrium solubility method. Water and a cosolvent (PEG or ethanol) were mixed in a vial volumetrically to form mixtures containing 0%, 10%, 20%, 30%, 40%, 50%, 60%, 70%, 80%, 90% and 100% of cosolvent. Excess drug was added directly into the mixed solvents under constant stirring at room temperature (25 ± 1 °C) for 24 h in order to obtain equilibrium. After 24 h of equilibration, samples were withdrawn, filtered (0.45 µm pore size) and diluted suitably. These samples were analyzed at 203 nm by UV spectroscopy (UV-2450, Shimadzu Corporation, Kyoto, Japan). All solubility experiments were conducted in triplicate.

### 2.3. Preparation of Phenytoin ODFs

Two film-forming polymers, PVA and HMP, were selected in concentrations of 1% *w*/*w* to prepare ODFs. ODFs were prepared by the solvent casting method. A polymeric solution was prepared by dissolving the film-forming polymer in a specific proportion in distilled water maintained at 60 °C and was stirred for 2 h with a magnetic stirrer. The obtained dispersion was then cooled down to room temperature, after which, the phenytoin powder or phenytoin cosolvent system with or without sodium starch glycolate (SSG) were added in the required quantity, as shown in Table 1. The films without cosolvent system (PVA-No and HMP-No) were used as control films to assess the effect of cosolvent system on film characteristics and drug release behaviors. The dispersion was gently stirred for 15 min and then left until all of the air bubbles disappeared. The solution was cast onto a plastic plate (square polyvinylchloride transparent plastic plate, 6 cm × 6 cm × 3.2 cm, Siam Packaging, Chiang Mai, Thailand), and then dried at 45 ± 2 °C for 24 h. The resultant film was cut into dimensions of 2 cm × 2 cm, in which 5 mg of phenytoin was included. Films with air bubbles, cuts or imperfections were excluded from the study.

### 2.4. Evaluation Parameters of Phenytoin ODFs

#### 2.4.1. Weight Variation

All films were evaluated for their weight variation. The weight variation was evaluated by using an analytical balance (MC-1 AC210S, Sartorius, Goettingen, Germany). Five replicates were conducted for each film. The average film weights (in g) with standard deviation were calculated.

#### 2.4.2. Thickness Uniformity

The thickness of each film was measured using an electronic digital thickness gauge (Deqing Syntek Electronic Technology Co., Ltd., Zhejiang, China) at three different points (left, middle and right) in the same position on each film (five replicates were conducted for each film), and the average film thickness (in mm) with standard deviation was calculated.

#### 2.4.3. Surface pH

The films were placed in contact with the electrode of a compact pH meter (LAQUAtwin-pH-22, Horiba Scientific, Kyoto, Japan), allowing equilibration for 30 s to determine the surface pH. The surface pH was determined for three films of each formulation, and the average film pH with the standard deviation was calculated.

#### 2.4.4. Mechanical Strength Test

The mechanical strength of the films was tested using a texture analyzer, TX.TA plus (Stable Micro Systems, Surrey, UK). An individual sample holder was constructed to facilitate the measurements of 2 cm × 2 cm film samples. The film was fixed on a plate with a cylindrical hole with a 9.0 mm diameter (the area of the sample holder hole was 63.56 mm^2^). A cylindrical stainless probe (2 mm in diameter) with a plane flat-faced surface was used (with a probe contact area of 3.14 mm^2^). The texture analyzer was adjusted for the probe’s forward movement at a velocity of 1.0 mm/s. Measurement started when the probe had contacted the sample surface (triggering force). The probe moved on at a constant speed until the film was torn. The breakage of films was detected when the peak force of texture profile analysis curve dropped. The applied force and the slope of the force–time curve were recorded. All of the experiments were conducted at room-temperature conditions (25 °C, 70% relative humidity). Five replicates were conducted for each film. The mechanical strength of the film was characterized by the puncture strength and Young’s modulus, which were calculated from the following Equations (1) and (2) [16,17]:

(1) $$Puncture\;strength=\;\frac{F_{max}}{A}$$

where $F_{max}$ is the rupture force (*N*) and A is the probe contact area (mm^2^).

(2) $$Young^{\prime }s\;modulus=\;\frac{Slope\;of\;force-time\;curve\;\left(N/s\right)}{Film\;thickness\;\times Probe\;speed}$$

### 2.5. In Vitro Disintegration Time

The disintegration time provided an indication of the disintegration characteristics of the film. Films with the required size (2 cm × 2 cm) were added into 3 mL of simulated salivary fluid pH 6.8, which was prepared according to the method of Guhmann et al. (2012) [18] at 37 ± 0.5 °C. The disintegration of films was detected by visual observation. The time taken by the films to break was measured as the in vitro disintegration time. All studies were performed in triplicate for each formulation. The optimum formulations in terms of the mechanical properties and disintegration time were selected for further experiments.

### 2.6. Determination of Moisture Content

The moisture content in the ODFs was determined using a moisture analyzer (MX50, A&D Co., Ltd., Tokyo, Japan). Approximately 1 g of the ODFs was accurately weighed onto a pre-dried aluminum pan and then heated at 105 °C until a stabilization of weight was achieved. The specification sheet of the moisture analyzer indicated that the reading unit for measurement was 0.01%. Measurements were made in triplicate. The moisture content was calculated by the following Equation (3):

(3) $$Moisture\;content\;\left(\%\right)=\;\frac{\left(wet\;weight-dry\;weight\right)}{wet\;weight}\times 100$$

### 2.7. Measurement of Film Porosity

The true density of films was determined using an Accupyc II 1340 gas pycnometer (Micromeritics, Norcross, GA, USA). The dry ODFs with known weights were placed in a sample cup of a known volume, which was then followed by purging with helium gas at 25–30 °C 10 times. The purge fill pressure and cycle fill pressure were set as 19.5 psig. The density of ODFs was calculated using the gas displacement method, and the film porosity was then calculated by the following Equation (4) [19]:

(4) $$Film\;porosity=\left(1-\frac{\left(mass\;of\;film/\left(film\;thickness\;\times area\right)\right)}{true\;density}\right)\times 100\%$$

### 2.8. Phenytoin Content

Three randomly taken films (size of 2 cm × 2 cm), which were selected based on the average weight results, were added into vials containing 5 mL of distilled water and set aside until the film dissolved completely. Then, 15 mL of ethanol was added and stirred at room temperature. The solution was withdrawn, filtered, diluted 50 times with ethanol and then analyzed by a UV spectrophotometer (UV-2450, Shimadzu Corporation, Kyoto, Japan) at 203 nm. The phenytoin contents were determined from the standard curve of phenytoin in ethanol, which demonstrated linearity with a high correlation coefficient (r^2^ = 0.9979). The following regression Equation (5) was obtained:

*y* = 0.0949*x* + 0.0052 (5)

where *y* is the absorbance and *x* is the concentration of phenytoin (µg/mL). The experiment was done in triplicate. The percentages of phenytoin content were calculated.

### 2.9. Differential Scanning Calorimetry (DSC)

The films were analyzed using a differential scanning calorimeter (DSC 1, Mettler-Telodo, Columbus, OH, USA). For differential scanning calorimetry (DSC) analyses, 5 mg of film samples were weighted and placed in an aluminum crucible (40 µL), which was immediately sealed. An empty sample pan was used as a reference. Film samples were heated from 0 to 350 °C at a rate of 10 °C/min. Nitrogen gas was used to flush the DSC cell at a flow rate of 200 mL/min to maintain an inert environment. Experiments were done in triplicate.

### 2.10. X-ray Diffraction (XRD)

The crystalline structures of the samples were analyzed at ambient temperature using an X-ray diffractometer (Rigaku Smartlab, Rigaku Corporation, Tokyo, Japan). One film sample was applied to the holder. A typical sample holder, which is a 2 mm thick aluminum plate with a 20 mm square hole in the center, was used. The measurement was conducted in reflectance. Diffraction patterns were collected from 5° to 60° with a 5°/min step and step size of 0.010. Samples were measured as received.

### 2.11. In Vitro Release Study

The in vitro release test of phenytoin ODFs was carried out in a glass cylindrical bottle (250 mL of capacity, a dimension of 70 mm and 143 mm in height) containing 100 mL Tris buffer pH 7.5 with 1% *w*/*v* sodium lauryl sulfate (SLS) [20]. The temperature was maintained at 37 ± 0.5 °C, with a rotation speed of 50 rpm. The cylindrical magnetic stirrer bar (20 mm length, Ø 6 diameter), which was made of polytetrafluoroethylene (PTFE), an inert and non-reactive material, was used to study the drug release profiles. The sample was withdrawn at different time intervals (1, 3, 5, 10, 15, 20, 30 and 60 min) and replaced with an equal volume of fresh dissolution medium, in order to maintain sink conditions throughout the experiment. Withdrawn samples were filtered, diluted and analyzed by a UV spectrophotometer (UV-2450, Shimadzu Corporation, Kyoto, Japan) at 208 nm (Figure S1). The phenytoin contents were determined from the standard curve of phenytoin in Tris buffer pH 7.5 with 1% *w*/*v* SLS, which demonstrated linearity with a high correlation coefficient (r^2^ = 0.9999). The regression equation of *y* = 0.0797*x* − 0.1053 was obtained, where *y* is the absorbance and *x* is the concentration of phenytoin (µg/mL). The average percentage released was calculated at each time interval. All of the dissolution runs were performed in triplicate.

### 2.12. Statistical Analysis

All data were presented as mean ± SD. A one-way ANOVA was used to evaluate the significance of differences at the significance level of a *p*-value < 0.05. Statistical analysis was performed using SPSS software version 16.0 (SPSS Inc., Chicago, IL, USA).

## 3. Results and Discussion

### 3.1. Phenytoin Solubility in Mixed-Solvent Systems

One proven method of enhancing the solubility of poorly water-soluble drugs is cosolvency or the addition of a water-miscible solvent, in which the drug has good solubility [21]. In the present study, two frequently used low-toxicity cosolvents, polyethylene glycol 400 (PEG) and ethanol, were used to investigate the effect of various cosolvents on the solubility of phenytoin. The solubility (mg/mL) of phenytoin in water–PEG and water–ethanol mixtures at room temperature with their dielectric constants (ε) are given in Table 2. The dielectric constants of water–PEG and water–ethanol mixtures were collected from the literature [22] and calculated using Equation (6):

ε_m_ = *f*_1_ε_1_ + *f*_2_ε_2_ (6)

in which ε_m_, ε_1_ and ε_2_ are the dielectric constants of mixed solvent, solvents 1 (cosolvent) and 2 (water), and *f*_1_ and *f*_2_ are the volume fractions of solvents 1 and 2, respectively [23].

As shown in Table 2, the solubility of phenytoin in water–PEG and water–ethanol mixtures considerably increased with the increase in the volume fraction of cosolvent and decrease in the dielectric constant of the mixture. Among all the mixed-solvent systems, the solubility of phenytoin was greatest in the mixed-solvent system composed of 80% *v*/*v* PEG and 20% *v*/*v* water, which presented the maximum solubility at 26.69 mg/mL. The solubility value of phenytoin in the water–PEG mixture was considerably higher when compared to that in the water–ethanol mixture. The increase in this value would indicate that phenytoin solubility varies according to the hydrophobicity of the cosolvent and polarity of mixed solvents. The phenytoin solubility increases with the decrease in the polarity of mixed solvents due to the effect of like-dissolve-like. PEG 400 is a cosolvent that is less polar than ethanol and has both a polar oxygen atom and a non-polar (CH_2_)_2_ group, in which the polar part is responsible for miscibility with water and the non-polar part is responsible for interacting with hydrophobic drug molecules, decreasing the hydrogen bond density of water and reducing the ability of the mixed-solvent system to squeeze out the non-polar drug [24,25]. Thus, adding PEG 400 as a cosolvent to water creates a less polar environment for the mixed solvent, resulting in more drug being able to solubilize in that mixture.

### 3.2. Film Preparation and Characterization

**Figure 1:**
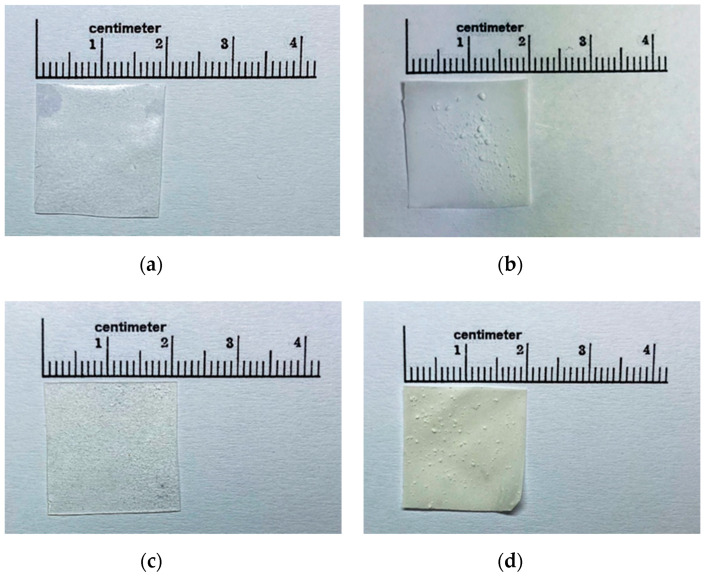
Gross appearance of polyvinyl alcohol (PVA)-S4 (**a**), PVA-No (**b**), high methoxyl pectin (HMP)-S4 (**c**) and HMP-No (**d**) phenytoin orally disintegrating films (ODFs).

According to the result of the phenytoin solubility study, a cosolvent system consisting of 6.76% *w*/*w* PEG 400, 1.12% *w*/*w* glycerin, and 1.12% *w*/*w* water of total formulation was developed to improve the solubility of phenytoin in the film formulation. Glycerin was selected as part of the cosolvent system, due to its safety profiles and its dielectric constant (ε = 42.5), which is close to the required dielectric constant (ε = 25.59). In addition, it is a commonly used excipient in pharmaceutical preparations. The overall risk of toxicity from glycerin found in pharmaceutical products is low, meaning that it can be used in large amounts. All the developed ODFs were prepared by a solvent casting method using PVA, HMP or a mixture of both as film-forming agents. Upon visual inspection, the films incorporated with the phenytoin cosolvent system were transparent, smooth and uniform, as shown in Figure 1a,c, whereas the films without a cosolvent system were opaque and showed a rough surface with the presence of drug particles on the surface of films (Figure 1b,d). Furthermore, the incorporation of the cosolvent system in the film formulation also provided a flexible film, because PEG 400 and glycerin also acted as plasticizers while the films without a cosolvent were more brittle and cracked. Additionally, when the cosolvent system was incorporated, we observed an increase in the thickness and weight of films. As shown in Table 3, compared to the films without a cosolvent system, the films with a cosolvent system exhibited a significantly higher weight and thickness (*p* < 0.05). The average thickness of films with the cosolvent system varied in the range of 0.20–0.25 mm, which is adequate for handling and use and falls within a typical range for buccal films (0.05–0.5 mm) [26]. Moreover, this study showed that the surface pH values of PVA, HMP and PH films were found to be in the range of 7.26–7.44, 3.71–4.90 and 5.24–5.64, respectively (Table 3). These differences in the surface pH values of films are attributed to differences in the pH values of PVA and HMP; PVA has a pH value of 5.0–6.5 (pK_a_ value of 17.69) [27], while HMP has a pK_a_ value of 3.55 [28]. The results indicated that the incorporation of acidic or alkaline drugs, polymers or solubilizers may influence the surface pH of films, leading to an acidic or alkaline microenvironment in the film–mucosa interface and causing mucosal irritation and damage [29]. According to the results obtained, the surface pH of PVA films was mostly close to neutral (pH 7), meaning that the PVA films are expected to suitable for use in the buccal cavity and not to cause any irritation to the buccal mucosa.

### 3.3. Mechanical Properties of Phenytoin ODFs

The mechanical parameters (puncture strength and Young’s modulus) of all developed films are shown in Table 4. In this study, the films with a cosolvent system showed significantly lower puncture strengths and Young’s moduli than those films without a cosolvent system (*p* < 0.05). The films with a cosolvent were very soft, flexible and able to cut up easily. While the films without a cosolvent, which were used as controls, were stiffer than those films with a cosolvent and showed their brittle nature as reported by many authors [30,31,32], thus, lowering their handling safety. In addition, there was no significant difference (*p* > 0.05) between the puncture strength and Young’s modulus values of the films with SSG and the films without SSG and no significant difference (*p* > 0.05) between the puncture strength and Young’s modulus values of the PVA films, HMP films and PH films. Thus, this study indicated that the presence of plasticizers such as PEG 400 and glycerin in the cosolvent system significantly altered the mechanical properties of films. The plasticizers can improve flexibility and reduce the brittleness of films by increasing the free volume between the polymer chain, which allows the polymer chains to move more freely, decreasing the intermolecular interaction between polymer chains and causing a reduction of mechanical resistance [33,34].

### 3.4. Disintegration Time

For the direct comparisons of in vitro disintegration times, the obtained disintegration times were normalized by thickness and are presented in Table 4. The results showed that, once the films were placed in the simulated salivary fluid, the films with a cosolvent system disintegrated in about 1–3 min, whereas the films without a cosolvent required only 20 s. Regarding the effect of thickness, the observed trend was that the disintegration time of the thinner films was faster, which was expected. Furthermore, the films without a cosolvent exhibited faster disintegration times, due to their higher porous structure. This was consistent with the results of film porosity study, which showed that the film porosity of PVA-No and HMP-No ODFs (71.38% ± 0.09% and 77.23% ± 0.07%, respectively) were higher than PVA-S4 and HMP-S4 ODFs (8.49% ± 0.09% and 6.41% ± 0.10%, respectively). The lower porosity values in PVA-S4 and HMP-S4 ODFs were attributed to their higher moisture contents and structural collapse. The films with a cosolvent system retained higher moisture contents than films without a cosolvent system due to the humectant properties of PEG and glycerin, which hold more water molecules, thus, lowering moisture evaporation during the film drying process at 45 °C. In addition, the high moisture content or the presence of PEG and glycerin reduced the glass transition temperature and promoted molecular mobility in the films which lead to various structural transformations at the molecular level, such as structural collapse. The structural collapse may cause by the stresses in the film due to the heating and removal of water from the polymeric matrix [35,36]. On the other hand, the films without a cosolvent system exhibited high porosity and low moisture content owing to their poor molecular mobility which was introduced by their rigid structures.

For the normalization of disintegration time with film thickness, we found that the addition of a superdisintegrant at optimum concentration decreased the normalized disintegration time. The optimum concentration of SSG (at 0.04% *w*/*w*) showed a faster disintegration time, but at a higher concentration, there was no further decrease in disintegration time. The mechanism of the disintegrant action of SSG is swelling or water wicking or a combination of these mechanisms [37], but in this study, SSG resulted in no additional acceleration of the disintegration at higher concentrations (at 0.08% *w*/*w*) and increased the disintegration time, possibly by resisting the breakup of films.

### 3.5. Phenytoin Loading Content

The theoretical content of phenytoin in all developed films with sizes of 2 cm × 2 cm was 5 mg. Considering this content as 100% of the loading content, the phenytoin loading contents in PVA-S4, PVA-No, HMP-S4 and HMP-No ODFs were found to be 100.27% ± 2.11%, 109.90% ± 2.64%, 104.17% ± 3.13% and 119.38% ± 4.12%, respectively. These results indicated that the films with a cosolvent system exhibited drug content uniformity within an acceptable range (95.0%–105.0%), as endorsed by the United States Pharmacopeia (USP) [20]. Meanwhile, the drug contents of films without a cosolvent system were found to be outside the limits of the pharmacopeia range. This may be due to the non-uniform distribution of the drug throughout the films and drug aggregation. According to our experiments, the non-uniformity distribution of drugs throughout the films without a cosolvent system and drug aggregation (especially in the middle of casting plate) resulted in high amounts of phenytoin in the middle of 6 cm × 6 cm film, and less amounts of drug near the edge of film. We also found that the edge of film was thinner than the middle and was ripped easily when the film was peeled off from the casting plate, while the area close to the middle of film without a cosolvent system can peel off from the casting plate without ripping. Thus, most of the films without a cosolvent system used in this study were cut from the area close to the middle. This may lead to very high masses of the films without a cosolvent system, and also very high and unexpected drug content in the films without a cosolvent system, because we selected the films for evaluating their drug content based on the average weight results. Thus, this study indicated that the preparation of ODFs without a cosolvent system (PVA-No, HMP-No ODFs) by the solvent casting method may not be suitable for drugs with a narrow therapeutic index or that are poorly water-soluble. However, for further study, many methods could be used to overcome the problem of the drug content uniformity of ODFs without a cosolvent system, such as the hot-melt extrusion technique and fused deposition modeling (FDM) 3D printing technology [38,39].

### 3.6. Differential Scanning Calorimetry (DSC)

**Figure 2:**
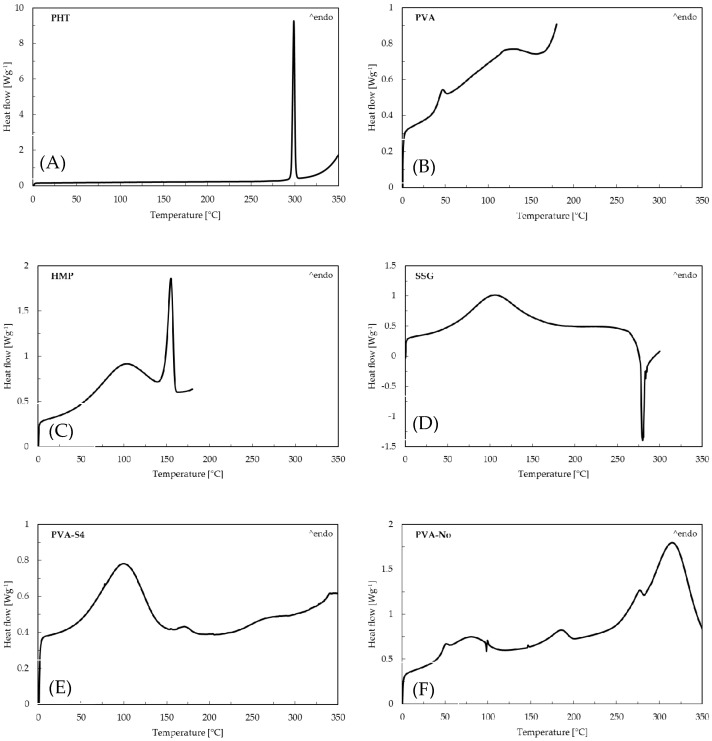

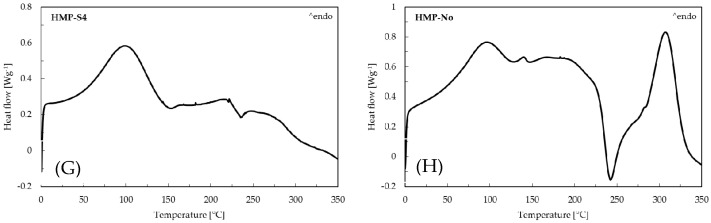
Differential scanning calorimetry (DSC) curves of (**A**) pure drug phenytoin, (**B**) PVA, (**C**) HMP, (**D**) SSG, (**E**) PVA-S4, (**F**) PVA-No, (**G**) HMP-S4 and (**H**) HMP-No ODFs.

Figure 2 illustrates the DSC thermograms of pure drug phenytoin (PHT), PVA, HMP, SSG, PVA-S4, PVA-No, HMP-S4 and HMP-No ODFs. According to this figure, a sharp endotherm peak corresponding to the melting point of crystalline phenytoin can be seen at 298.83 °C, which matches the values in the literature [40]. The PVA-S4 and HMP-S4 ODFs did not exhibit any sharp endothermic peak at nearly 298.83 °C. The absence of endothermic phenytoin could result from the drug dissolving in polymer matrices during the heating cycle, because the XRD results showed the diffraction of PHT in all preparations. On the other hand, an endothermic event was observed in the DSC thermograms of PVA-No and HMP-No ODFs at 314.5 and 307.17 °C, respectively. This could be due to the degradation of the polymers [39,41]. Furthermore, all developed films also exhibited a broad endothermic peak in the region of 60–125 °C, which corresponded to the evaporation of water [42]. This was consistent with the moisture content results, showing that there was water retained in the films. The moisture contents in PVA-S4, PVA-No, HMP-S4 and HMP-No ODFs were found to be 12.47% ± 0.53%, 6.01% ± 1.04%, 15.83% ± 0.06% and 7.35% ± 2.13%, respectively. Thus, the results indicated that PVA-S4 and HMP-S4 ODFs showed a much higher rate of water evaporation than the PVA-No and HMP-No ODFs. The higher moisture contents of PVA-S4 and HMP-S4 ODFs were likely caused by the PEG and glycerin in the formulations. Additionally, the high moisture content reduced the glass transition temperature and promoted molecular mobility in the films [43]. This may have increased the recrystallization tendency of PHT in the aging PVA-S4 and HMP-S4 ODFs films.

### 3.7. X-ray Diffraction (XRD) Studies

**Figure 3:**
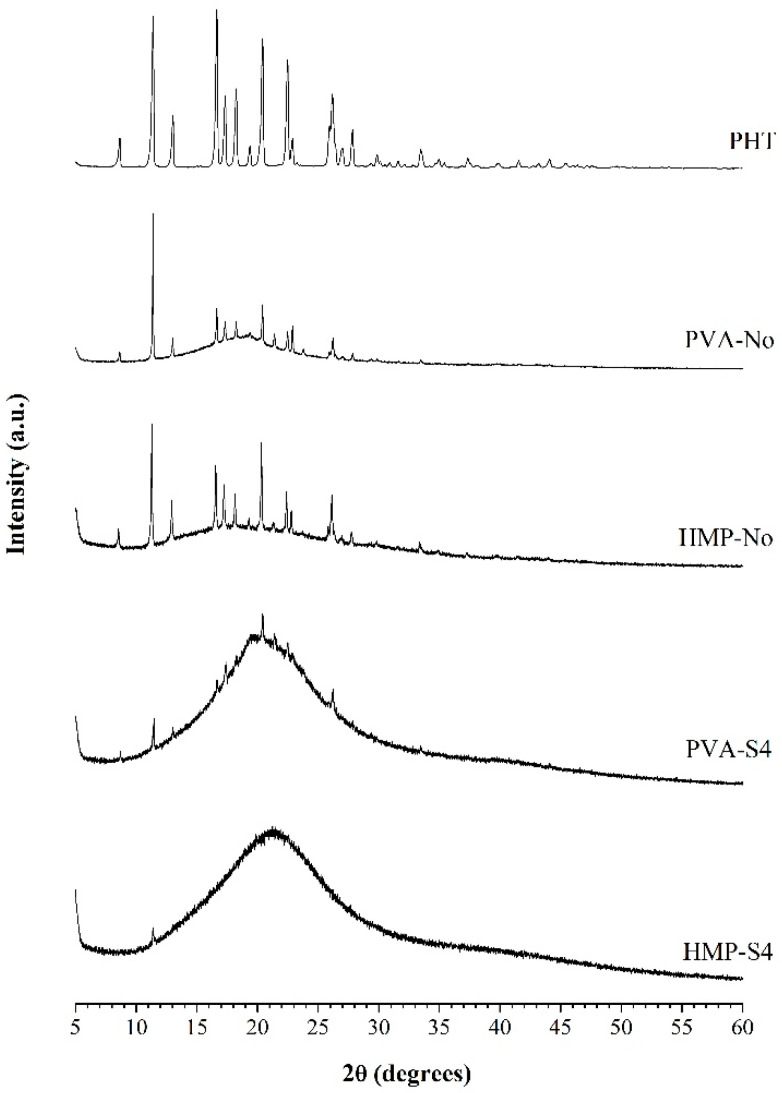
X-ray diffraction patterns of PHT (phenytoin), PVA-No, HMP-No, PVA-S4 and HMP-S4 ODFs.

Figure 3 shows the X-ray diffraction patterns of PVA-No, HMP-No, PVA-S4, HMP-S4 ODFs and the pure PHT drug, which was used as a reference. For the pure PHT drug, the X-ray diffractogram revealed various peaks over the entire scan range. The sharp and highly intense peaks at 11.40°, 13.04°, 16.65°, 17.34°, 18.25°, 20.42°, 22.48° and 26.17° confirmed the crystalline nature of the pure PHT drug. This X-ray diffraction pattern of PHT had similar peak positions to the observed peaks in the literature [44]. All film preparations presented diffraction peaks of PHT. The diffraction patterns presented in PVA-No and HMP-No films corresponded to the films prepared without a cosolvent in their formulations. In those samples, a slight decrease of diffraction peaks at 11.40°, 13.04°, 16.65°, 17.34°, 18.25°, 20.42°, 22.48° and 26.17° was observed. Moreover, the X-ray diffraction pattern of PVA-No and HMP-No films was similar to the pure PHT drug, which suggested that the drug is in a crystalline state in these films. However, a strong reduction of crystallinity occurred when the cosolvent system in the film formulations was increased, resulting in a considerable decrease of diffraction peaks in the X-ray diffraction pattern. As illustrated in the X-ray diffraction patterns of PVA-S4 and HMP-S4 ODFs, some diffraction peaks at 11.47°, 13.04°, 16.67°, 17.39°, 18.28°, 20.41°, 22.51° and 26.21° for PVA-S4 ODFs and 11.38° for HMP-S4 ODFs were observed. However, they were significantly broadened, and a decrease of peak intensities appeared, which indicated that the drug was converted to a partial amorphous form. This study revealed the transformation of a crystalline material to a disordered amorphous form by the cosolvent solubilization enhancing technique for poorly water-soluble PHT drugs. In addition, the amorphous state has long been recognized as a way to increase the free energy and the apparent aqueous solubility of poorly water-soluble pharmaceuticals [45,46].

### 3.8. In Vitro Phenytoin Release Studies

**Figure 4:**
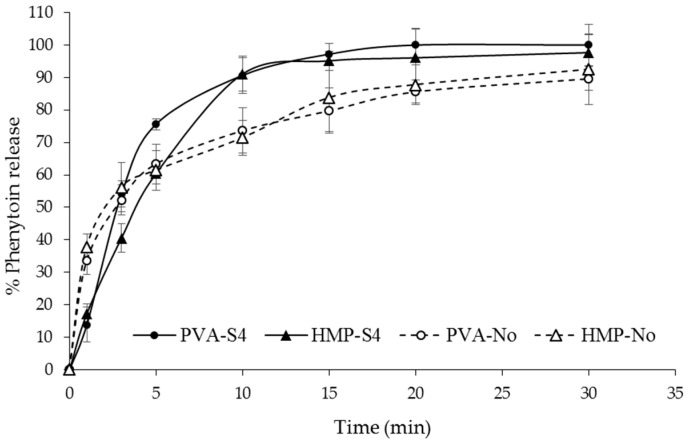
In vitro phenytoin release profile of PVA-S4, HMP-S4, PVA-No and HMP-No ODFs in Tris buffer pH 7.5 with 1% *w*/*v* sodium lauryl sulfate (SLS) at different time intervals from 0 to 30 min.

The plots of the percentage of cumulative drug release versus time of the selected ODFs are plotted and shown in Figure 4. The dissolution rate was found to differ with the presence of a cosolvent in the film formulation. PVA-No and HMP-No films showed significantly faster release at 1 min than PVA-S4 and HMP-S4 films, which may have been due to the dissolution of drug particles on the surface of PVA-No and HMP-No films, their high porous structure and their fast disintegration ability within 1 min, as a result of which, the films were turned into small pieces, thus, creating more pores and allowing the drug to diffuse out of the films. PVA-No and HMP-No ODFs showed a higher porosity in comparison to PVA-S4 and HMP-S4 ODFs. More than 75% of drug release was obtained from the PVA-S4 film within 5 min, whereas the other films (PVA-No, HMP-S4 and HMP-No) exhibited around 60% drug release. For PVA-S4 and HMP-S4 films, almost 100% drug release was obtained within 15 min, while PVA-No and HMP-No films may need more than 30 min to obtain 100% drug release. From this dissolution profile, it can be noted that the dissolution of poorly water-soluble drugs, such as phenytoin, in the form of ODF can be enhanced by using a cosolvent system in the film formulation. Generally, solubility depends on the formation of intermolecular hydrogen bonds between solvent molecules and the solute molecules. The crystalline form is more stable than the amorphous form and has lower energy at the molecular level, with stronger bonding between molecules that require higher energy to break [47,48]. Thus, using an amorphous form of the drug is an excellent option to improve its low aqueous solubility, since both the dissolution rates, as well as the apparent solubility are enhanced when converting a crystalline form to its amorphous counterpart [49]. Moreover, it was observed that, in the formulation containing PVA as a polymer (PVA-S4), the drug release at 3–5 min was found to be significantly faster than the film containing HMP as a polymer (HMP-S4), even though there was less amorphous drug in PVA-S4 as compared with HMP-S4, according to the XRD results (Figure 3). This may be due to the larger amount of free hydroxyl groups in the polymeric matrix of PVA than in HMP and the higher solubility of PVA. PVA has a solubility in water of 110 mg/mL [50], whereas HMP has a solubility in water of 13 mg/mL [51]. Additionally, those hydroxyl groups of PVA interact with water molecules through hydrogen bonds, allowing the formation of hydrogen interactions between the film and water molecules [52]. However, the solubility of PVA depends on its degree of hydrolysis, which is inversely proportional to its solubility [53]. In PVA with a lower degree of hydrolysis (as used in our study), inter- and intra- molecular H-bonds are reduced due to steric hindrance from a large quantity of hydrophobic acetate groups. This helps to increase the interactions between PVA molecules and water molecules, thus boosting solubility [54]. Accordingly, the results indicated that it is more important to use more low-molecular excipients (PEG400, glycerin, water) and more hydrophilic polymer (PVA) in the formulation than to achieve a fully amorphous state of the API.

## 4. Conclusions

In this study, orally disintegrating films of phenytoin were successfully formulated by means of a cosolvent system using the solvent casting method. The incorporation of a cosolvent system in the film formulation improved the flexibility of films and reduced drug crystallinity, which appeared to enhance the drug solubility and drug release rate. However, the films with a cosolvent system disintegrated slower than those without a cosolvent system, and the cosolvent system lost its advantage from the perspective of disintegration time. Among all developed formulations, the film composed of 1% *w*/*w* PVA as a polymer, 0.04% *w*/*w* SSG as a superdisintegrant and PEG, glycerin and water as cosolvents (PVA-S4 film) was identified as the best formulation. It showed an acceptable disintegration time of less than 3 min along with a good dissolution profile and physical and mechanical characteristics, such as appearance, surface pH, smoothness and flexibility. This developed ODF formulation could potentially be applied to load other poorly water-soluble drugs. Moreover, this study indicated that the cosolvent solubilization technique is an interesting technique for stabilizing the amorphous phase and enhancing the apparent solubility of a poorly water-soluble drug in an ODF formulation. However, this phenytoin ODFs needs further development, especially regarding the capability of loading large amounts of drug, to achieve the dose required for the treatment of epilepsy and experimental kinetic data with different kinetic models, in order to set the drug release mechanism.

## Figures and Tables

**Table 1:** Composition of different film casting solutions.

Formulation Code	Polymer (%*w*/*w*)		SSG (%*w*/*w*)	Phenytoin (%*w*/*w*)	Cosolvent System (%*w*/*w*)			Water
	PVA	HMP			PEG	Glycerin	Water	qs to
PVA-S0	1	-	-	0.45	6.76	1.12	1.12	100
PVA-S4	1	-	0.04	0.45	6.76	1.12	1.12	100
PVA-S8	1	-	0.08	0.45	6.76	1.12	1.12	100
PVA-No	1	-	-	0.45	-	-	-	100
HMP-S0	-	1	-	0.45	6.76	1.12	1.12	100
HMP-S4	-	1	0.04	0.45	6.76	1.12	1.12	100
HMP-S8	-	1	0.08	0.45	6.76	1.12	1.12	100
HMP-No	-	1	-	0.45	-	-	-	100
PH-S0	0.5	0.5	-	0.45	6.76	1.12	1.12	100
PH-S4	0.5	0.5	0.04	0.45	6.76	1.12	1.12	100
PH-S8	0.5	0.5	0.08	0.45	6.76	1.12	1.12	100

Note: PVA: polyvinyl alcohol film; HMP: high methoxyl pectin film; PH: polyvinyl alcohol and high methoxyl pectin film; S0, S4, S8: the film without superdisintegrant sodium starch glycolate (SSG) and the film containing 0.04 or 0.08% *w*/*w* of SSG No: the film without cosolvent.

**Table 2:** Solubility profiles of phenytoin in water–polyethylene glycol (PEG) and water–ethanol mixtures of differing dielectric constants.

Cosolvent (%*v*/*v*)	Water (%*v*/*v*)	Dielectric Constant		Solubility of Phenytoin (mg/mL)	
		Water–PEG	Water–Ethanol	In Water–PEG	In Water–Ethanol
100	0	12.40	24.30	26.52	18.54
90	10	19.00	29.71	26.36	18.16
80	20	25.59	35.11	26.69	15.77
70	30	32.19	40.52	21.21	11.11
60	40	38.78	45.92	10.17	7.62
50	50	45.38	51.33	3.91	4.86
40	60	51.98	56.74	2.60	2.19
30	70	58.57	62.14	0.24	1.42
20	80	65.17	67.55	ND	ND
10	90	71.76	72.95	ND	ND
0	100	78.36	78.36	ND	ND

**Table 3:** Weight, thickness and surface pH of phenytoin orally disintegrating films (ODFs).

Film	Weight (g ± SD)	Thickness (mm ± SD)	Surface pH
PVA-S0	0.1090 ± 0.0057 ^a^	0.213 ± 0.008 ^a^	7.26 ± 0.11 ^a^
PVA-S4	0.1063 ± 0.0051 ^a^	0.203 ± 0.039 ^a^	7.47 ± 0.17 ^a^
PVA-S8	0.1083 ± 0.0019 ^a^	0.232 ± 0.012 ^a^	7.52 ± 0.18 ^a^
PVA-No	0.0141 ± 0.0011 ^b^	0.084 ± 0.042 ^b^	7.44 ± 0.10 ^a^
HMP-S0	0.1106 ± 0.0023 ^a^	0.226 ± 0.005 ^a^	4.90 ± 0.32 ^b^
HMP-S4	0.1079 ± 0.0038 ^a^	0.234 ± 0.010 ^a^	4.36 ± 0.12 ^b^
HMP-S8	0.1151 ± 0.0070 ^c^	0.244 ± 0.010 ^a^	4.67 ± 0.05 ^b^
HMP-No	0.0160 ± 0.0015 ^b^	0.106 ± 0.008 ^b^	3.71 ± 0.12 ^c^
PH-S0	0.1170 ± 0.0018 ^c^	0.238 ± 0.009 ^a^	5.24 ± 0.02 ^d^
PH-S4	0.1165 ± 0.0007 ^c^	0.242 ± 0.003 ^a^	5.31 ± 0.09 ^d^
PH-S8	0.1239 ± 0.0047 ^c^	0.251 ± 0.017 ^a^	5.64 ± 0.02 ^d^

For each test, means with the same letter are not significantly different. Thus, means with different letters, e.g., “a” or “b”, are statistically different (*p* < 0.05).

**Table 4:** Mechanical properties and disintegration time of phenytoin ODFs.

Film	Puncture Strength (N/mm^2^)	Young’s Modulus (N/mm^2^)	Disintegration Time (min)	Normalized Disintegration Time (min)
PVA-S0	0.15 ± 0.02 ^a^	4.12 ± 0.61 ^a^	2.68 ± 0.23 ^a^	3.15 ± 0.26 ^a^
PVA-S4	0.14 ± 0.01 ^a^	2.05 ± 0.22 ^a^	1.44 ± 0.26 ^b^	1.78 ± 0.32 ^b^
PVA-S8	0.14 ± 0.01 ^a^	2.12 ± 0.16 ^a^	2.38 ± 0.25 ^a^	2.57 ± 0.27 ^a^
PVA-No	2.47 ± 0.20 ^b^	203.88 ± 37.99 ^b^	0.33 ± 0.04 ^c^	0.99 ± 0.14 ^c^
HMP-S0	0.33 ± 0.03 ^a^	19.11 ± 2.32 ^a^	3.35 ± 0.62 ^d^	3.72 ± 0.69 ^d^
HMP-S4	0.39 ± 0.05 ^a^	12.14 ± 1.36 ^a^	1.62 ± 0.06 ^b^	1.74 ± 0.07 ^b^
HMP-S8	0.43 ± 0.04 ^a^	12.84 ± 1.20 ^a^	2.14 ± 0.71 ^a^	2.19 ± 0.73 ^b^
HMP-No	1.82 ± 0.36 ^c^	120.86 ± 20.16 ^b^	0.33 ± 0.01 ^c^	0.76 ± 0.01 ^c^
PH-S0	0.30 ± 0.04 ^a^	14.35 ± 1.01 ^a^	2.75 ± 0.04 ^a^	2.90 ± 0.05 ^a^
PH-S4	0.29 ± 0.02 ^a^	7.02 ± 1.02 ^a^	2.08 ± 0.24 ^a^	2.15 ± 0.24 ^b^
PH-S8	0.30 ± 0.03 ^a^	7.05 ± 1.62 ^a^	2.54 ± 0.09 ^a^	2.54 ± 0.09 ^a^

For each test, means with the same letter are not significantly different. Thus, means with different letters, e.g., “a” or “b”, are statistically different (*p* < 0.05).

## Supplementary Materials

The following are available online at https://www.mdpi.com/2077-0375/10/12/376/s1, Figure S1: UV spectra of phenytoin.
