# Supplementary material for: Formulation of Orally Disintegrating Films as an Amorphous Solid Solution of a Poorly Water-Soluble Drug
Source: Membranes (Basel). 2020 Nov 27;10(12):376. doi: 10.3390/membranes10120376 (PMC7759778; doi:10.3390/membranes10120376)
Supplement: Supplementary file 1 [file membranes-10-00376-s001.pdf]

membranes

Supplementary Materials: Formulation of Orally Disintegrating Films as an Amorphous Solid Solution of a Poorly Water-Soluble Drug

Pattaraporn Panraksa [1], Pratchaya Tipduangta [1], Kittisak Jantanasakulwong [2,3] and Pensak Jantrawut [1,3,*]

[1] Department of Pharmaceutical Sciences, Faculty of Pharmacy, Chiang Mai University, Chiang Mai 50200, Thailand; pattaraporn.prs@gmail.com (P.P.); ptipduangta@gmail.com (P.T.)
[2] Division of Packaging Technology, School of Agro-Industry, Faculty of Agro-Industry, Chiang Mai University, Chiang Mai 50100, Thailand; jantanasakulwong.k@gmail.com
[3] Cluster of Agro Bio-Circular-Green Industry (Agro BCG), Chiang Mai University, Chiang Mai 50100, Thailand
* Correspondence: pensak.amuamu@gmail.com, pensak.j@cmu.ac.th; Tel.: +6653944309 (P.J.)

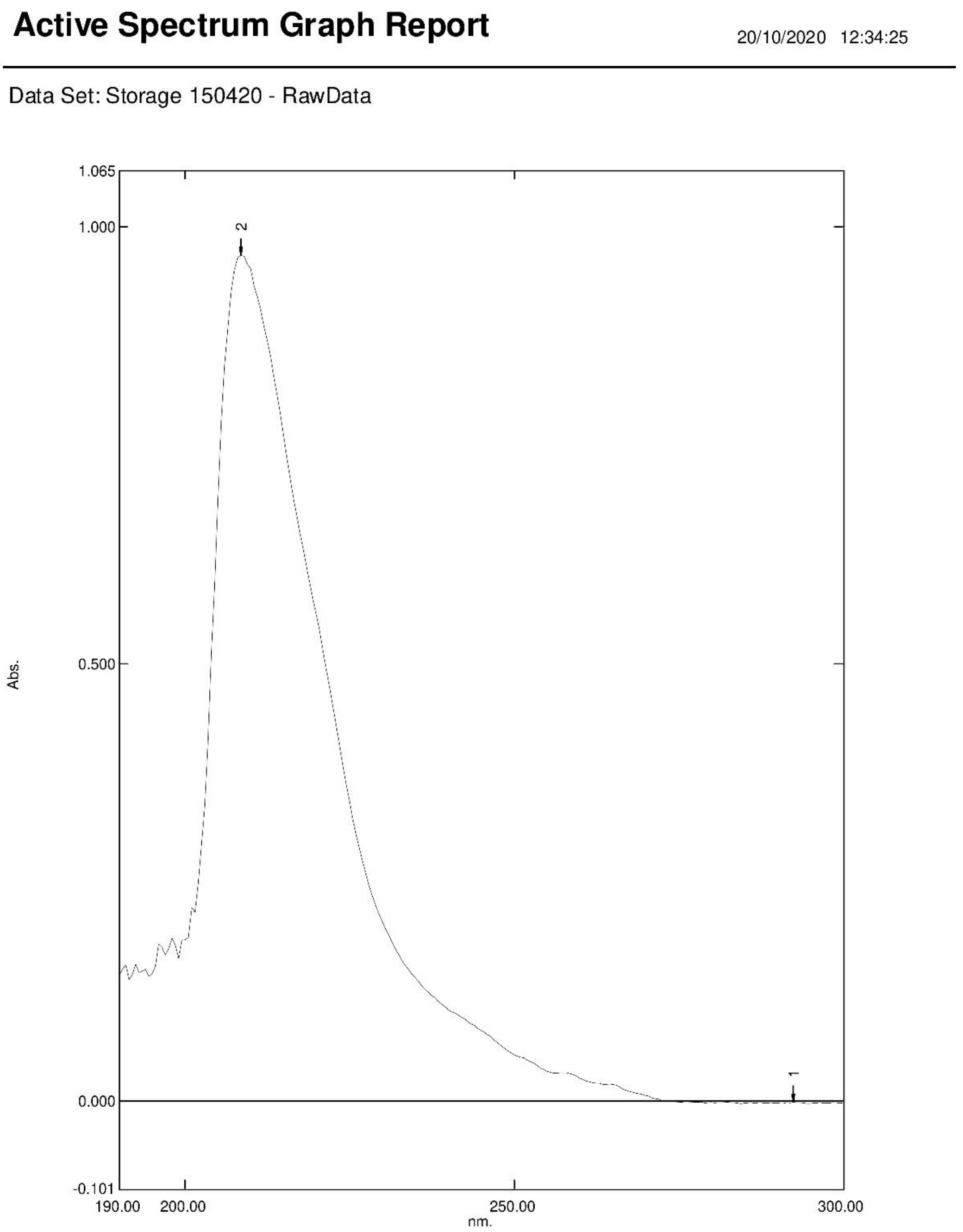

Figure S1. UV spectra of phenytoin.
